# An uncommon case of lymphoplasmacytic lymphoma in cerebellopontine angle region: Case report with a literature review

**DOI:** 10.1097/MD.0000000000004627

**Published:** 2016-08-26

**Authors:** Chengrui Yan, Xiangyi Kong, Lanshu Yang, Wenbin Ma

**Affiliations:** Department of Neurosurgery, Peking Union Medical College Hospital, Chinese Academy of Medical Sciences, No. 1 Shuaifuyuan, Beijing, P. R. China.

**Keywords:** cerebellopontine angle region, differential diagnosis, lymphoplasmacytic lymphoma

## Abstract

In the central nervous system, cerebellopontine angle (CPA) lymphomas are rare; few cases have been reported. Lymphoplasmacytic lymphoma (LPL) in the CPA is rarer still, and often misdiagnosed as acoustic neuroma.

We report a rare case of CPA LPL—a challenging diagnosis guided by clinical presentations, radiological signs, and postoperative pathological test.

A 43-year-old woman presented with headaches. Her magnetic resonance imaging revealed an abnormal homogeneously enhancing mass in the left CPA. We present detailed analysis of her disease and review relevant literature.

When surgically treated, her specimen showed a typical LPL histopathology pattern. After surgery, the patient's symptoms improved greatly, and she received chemotherapy.

Despite its rarity, LPL should be considered in differential diagnoses of CPA lesions that mimic acoustic neuromas.

## 1 Introduction

Primary central nervous system (CNS) lymphoma is a very rare aggressive non-Hodgkin disease that originates in CNS (brain, leptomeninges, spinal cord, or eyes) without evident involvement of other systemic locations. They account for less than 1% of all intracranial tumors but have apparently increased over the last 2 decades in both immunocompromised and immunocompetent patients.^[1]^ They may be solitary or multiple, with a predilection for the corpus callosum, basal ganglia, thalami, and paraventricular region.^[1]^ Cerebellopontine angle (CPA) lymphomas are rare and only a handful of cases have been reported so far. Lymphoplasmacytic (or lymphoplasmacytoid) lymphoma (LPL) is an uncommon mature B-cell lymphoma that usually involves the bone marrow and, less commonly, the lymph nodes and spleen.^[2]^ LPLs in the CNS are uncommon, and rarer still in the CPA.

Here, we report an uncommon solitary primary LPL in the CPA, and discuss its treatment and differential diagnoses. Although a few CPA lymphomas have been reported previously, this is the first reported case of a CPA LPL, to our knowledge.

### 1.1 Case presentation

**Figure 1:**
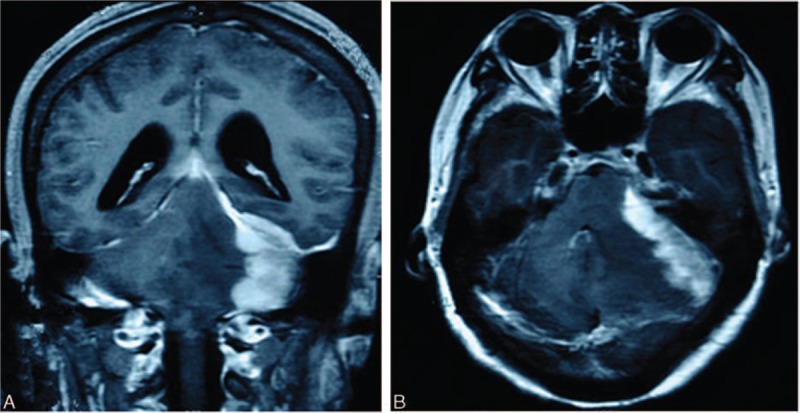
Contrast-enhanced MRI of the brain showed an enhanced mass, measuring 5 cm × 5 cm × 3.5 cm in the right CPA.

A 43-year-old woman was admitted to our hospital complaining of continuous frontal and occipital headaches for 4 months, and a left hearing loss for 2 months. One week before admission, she began vomiting for about 5 times a day. She had no significant medical background or family history. No lymphadenopathy or organomegaly were found upon general physical examination. Skull, bone, cardiac, chest, and per rectal examinations revealed no abnormalities. She was oriented and conscious. Visual fields and acuity was normal. Neurological examinations revealed normal motor function and sensation in all four limbs. No obvious changes in superficial or deep tendon reflexes were detected. Ataxia and pathological signs were absent. Routine hematological test results were all normal and the serological HIV test was negative. However, a brain magnetic resonance imaging (MRI) showed a homogeneously enhancing mass in the left CPA, measuring 5 cm × 5 cm × 3.5 cm; neuritis or a CPA tumor was suspected (Fig. 1). The tumor showed relatively equal signal intensities on T2-weighted imaging (T2WI) and T1-weighted imaging (T1WI), extending slightly to the left temporal pole meninges. The mass also mildly compressed the cerebellum, the adjacent brain stem, the aqueduct of Sylvius, and the fourth ventricle. The MRI revealed cerebellar swelling, dilation of supratentorial ventricles system, and interstitial edema. Along with her clinical history, these findings implied an acoustic neuroma in the left side.

The patient underwent a left retromastoid craniectomy and a tumor resection. During surgery, a well-defined left CPA lesion extending to the left internal auditory canal was seen. It was against the petrous bone and tentorium cerebelli, with abundant vascularization and relatively clear boundaries. The tumor was extirpated as multiple pieces of friable, gray, and firm tissues, which were fixed in 10% paraformaldehyde and embedded in paraffin. Microscopically, the tissues were a mixture of cancerous lymphocytes and plasma cells. Immunohistochemical tests showed the tissues to be positive for CD38, CD20, CD38, and CD79α, and negative for AE1/AE3, Bcl-6, CD3, CD10, glial fibrillary acidic protein, neuron-specific enolase, and Syn. The positive rate for Ki-67 was 10%.

**Figure 2:**
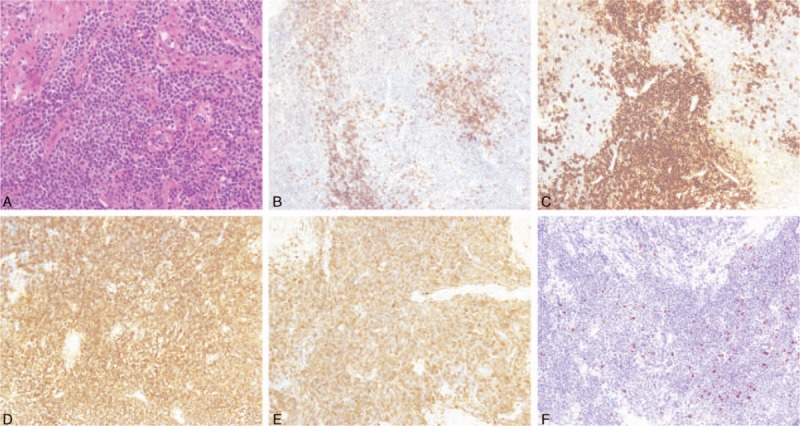
Postsurgical pathological and immunohistochemical examination confirmed the diagnosis as LPL.

Pathological examination confirmed the diagnosis of LPL (Fig. 2). The patient then underwent a bone marrow biopsy and a computed tomography (CT) scan of the entire chest, abdomen, and pelvis. No abnormalities were found. According to the Ann Arbor–Cotswald staging system for lymphomas, this case of CPA LPL was classified as Stage I, indicating that the cancer was located in a single region.

**Figure 3:**
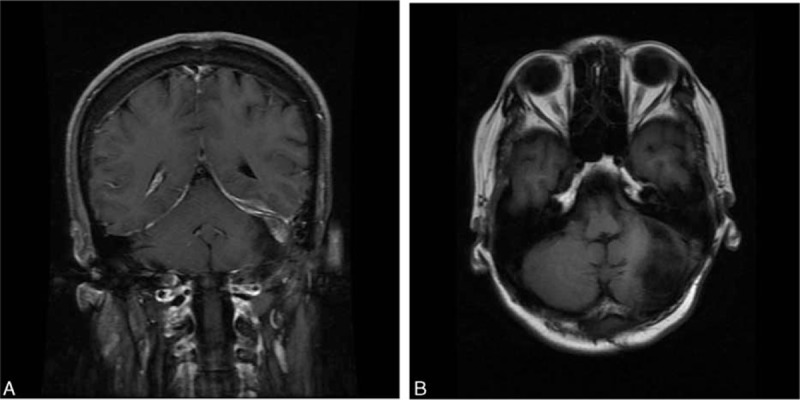
Half a year after the surgery, a MRI was performed, showing no recurrence.

One week after the surgery, the patient's headache and hearing loss were much relieved. Postoperative MRI showed that the CPA tumor was totally removed, and adjacent structures were thoroughly decompressed. She was then transferred to the hematology department for chemotherapy. The chemotherapy protocol was the cyclophosphamide, vincristine, and prednisone regimen along with rituximab. After 6 cycles of chemotherapy, her Karnofsky Performance Scale score was 95. An MRI performed half a year after the surgery showed no recurrence (Fig. 3).

## 2 Discussion

A few rare tumors can occur in the CPA. After excluding cranial nerve schwannomas (1.4%), cholesteatoma (2.4%), meningioma (3.1%), and acoustic neuromas (91.3%) from a series of 1354 CPA tumors, 25 uncommon tumors remained, including lipomas, metastatic tumors, gliomas, hemangiomas, hemangioblastomas, arachnoid cysts, dermoids, and a teratoma.^[3]^ In a series of 32 nonacoustic CPA tumors, hemangiosarcoma was also recorded.^[3]^

Primary central nervous system lymphomas (PCNSLs) are uncommon tumors that account for approximately 0.8% of lymphomas at all sites and 0.3% to 1.5% of all intracranial tumors.^[4]^ They may occur in both immunocompromised and immunocompetent individuals.^[4]^ For immunocompetent patients, the mean age at diagnosis of PCNSL is 55 years; for AIDS patients, the mean age is 31 years. The female: male ratio is 2: 3. PCNSLs often occur in thalami, periventricular region, corpus callosum, and basal ganglia.^[5]^ However, only 15 cases of PCNSLs at the CPA have been reported in the literature.^[6]^ Almost all PCNSLs are aggressive neoplasms of diffuse large B-cell lymphoma type; low-grade malignant lymphomas such as peripheral T-cell, Burkitt lymphoma, marginal mantle cell lymphoma, and LPL are rare.^[7]^

LPL is a kind of non-Hodgkin lymphoma with low-grade malignancy, and composed of plasma cells, plasmacytoid lymphocytes, and small B cells. Its incidence is approximately 3% to 4.5% per year worldwide.^[8]^ Most patients demonstrate increased monoclonal serum IgM, and a few have elevated IgA or IgG.^[9]^ LPL usually involves the bone marrow and, less commonly, the spleen and lymph nodes. The 2008 World Health Organization (WHO) classification defined Waldenström macroglobulinemia (WM) as a clinicopathologic entity associated with an IgM monoclonal gammopathy and marrow involvement, which is the commonest manifestation of LPL.^[10]^ The pathogenesis of LPL is incompletely understood. However, deletions of 6q21 have been identified as a common site of chromosome loss in patients with WM.^[11]^ These findings suggest that *MYD88* mutations that lead to the activation of NF-kB family transcription factors are pivotal role in the pathogenesis of LPL.^[2,12]^ Chronic infection with HIV and hepatitis C virus (HCV) has also been implicated as risk factors.

To our knowledge, only 24 cases of primary CNS LPL have been reported previously,^[13–16]^ of which 9 as well as the present one included detailed information (Table 1). Our case is the first reported case of LPL originating in the CPA.

**Table 1:**
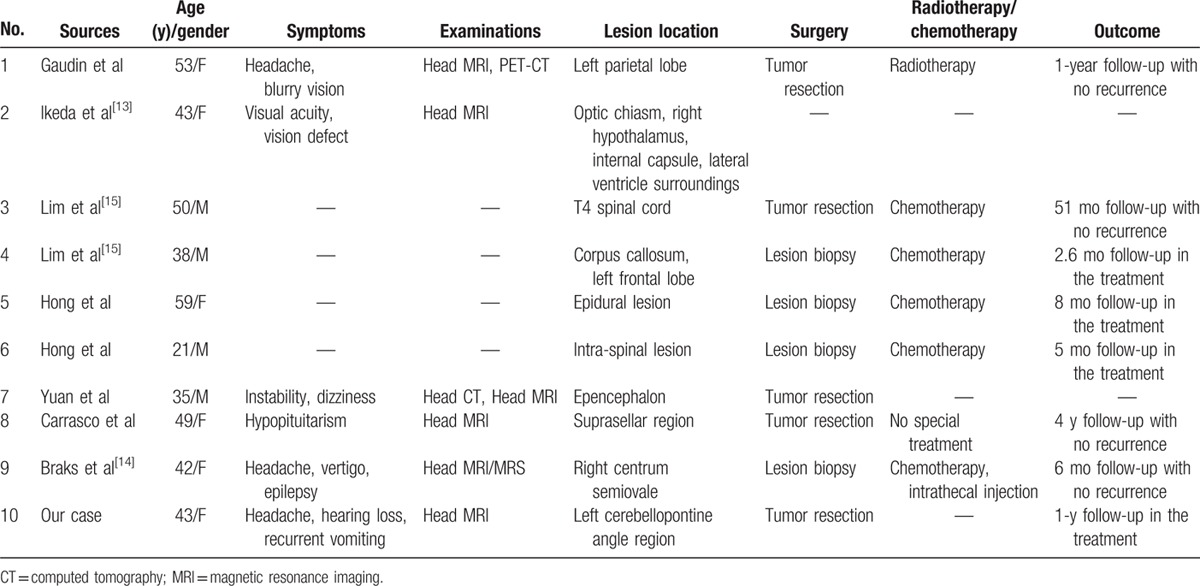
Clinical features of the reported cases of primary CNS LPL.

The average onset age of primary CNS LPL is 43.3 years, and a high proportion of patients are young or middle-aged (Table 1). The brain parenchyma and meninges are the most commonly involved sites. Occupying lesions are most commonly found through head MRIs.^[9]^ Clinical presentations of patients with CNS LPL vary; the most common but atypical features include focal symptoms and intracranial hypertension; however, typical WM presentations such as infiltration of other organs and gammopathy are rare.^[17]^ Solitary CPA lymphoma must be distinguished from acoustic neurilemomas (which account for 80–90% of all CPA tumors), meningiomas (2–3%), and epidermoid tumors (1–2%).^[18]^ Other rarer CPA tumors include facial nerve neurilemomas, arachnoid cysts, paraganglioma of the glomus jugulare, lipomas, hemangiomas, angioblastoma, choroid plexus papillomas, teratoma, craniopharyngioma, gliomas, liposarcoma, chondrosarcoma, medulloblastoma, metastatic tumors, primary epidermoid carcinomas, endodermal sinus tumors, and malignant schwannoma with rhabdomyoblastic differentiation. Signs, symptoms, and tests—especially imaging examinations—of some common differential diagnoses are listed in Table 2. Surgical intervention, including a biopsy for a pathological diagnosis, is highly recommended. Some studies have also indicated that 13.3% to 16% patients had a positive result from cerebrospinal fluid cytological examinations.^[17]^ The CPA LPL immunophenotype is sometimes similar to other small B-cell lymphoid neoplasms, such as marginal mental cell lymphoma; thus, testing for serum immunoglobulin and gene mutation can be useful.

**Table 2:**
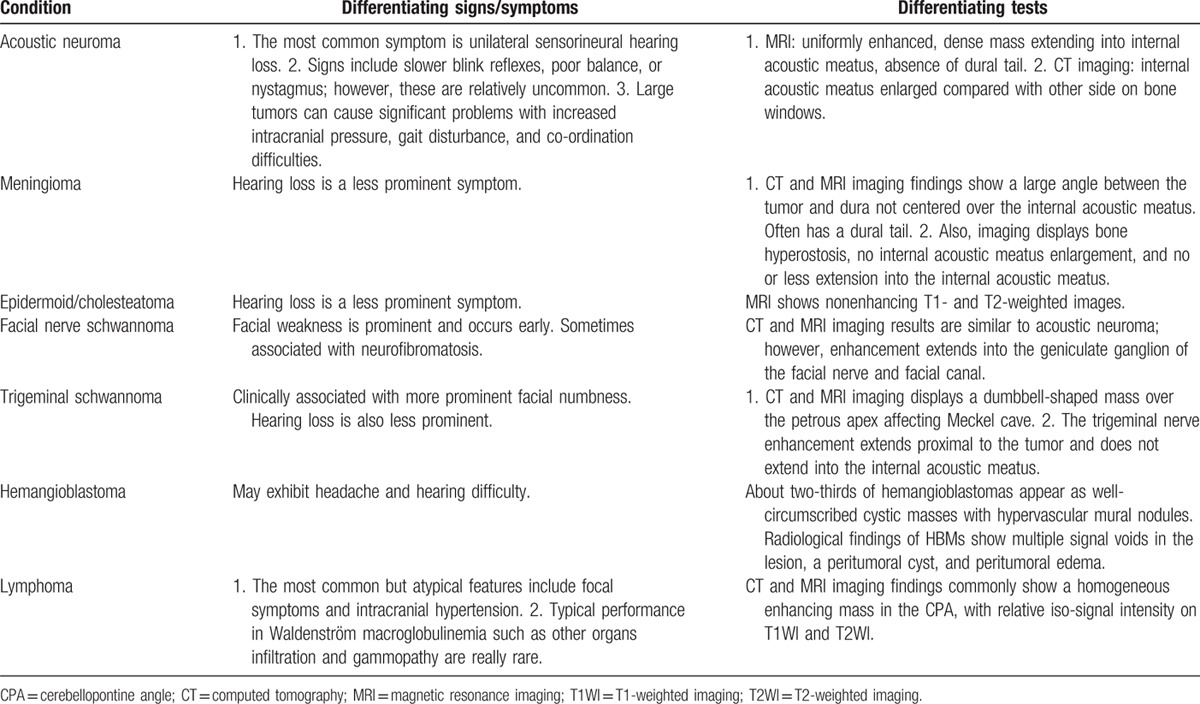
Differential diagnoses of CPA lesions.

The predominant treatments for primary CNS LPL are surgery, radiotherapy, chemotherapy, and molecular targeted therapy.^[16]^ As the cases are few, no specialized guideline has yet been developed. The chemotherapy regimen is cyclophosphamide + doxorubicin + vincristine + prednison (CHOP) or cyclophosphamide, vincristine, and prednisone, with rituximab—a monoclonal antibody widely used against B cell lymphoma.^[19]^ Patients who received rituximab along with CHOP reportedly had significantly higher rates of overall response than patients who only received the traditional regimen.

The positive rate of Ki-67 in LPL is generally less than 20% (∼10% in our case), which leads to a benign prognosis. For asymptomatic patients, regular follow-up alone is recommended.^[20]^ No accurate prognostic data are available for primary CNS LPL because of the scarcity of case reports. LPL patients with no special treatment are reported to have a median survival of longer than 5 years; their 10-year survival rate is around 70% to 75%.^[16,21]^ No deaths were reported among the 7 patients with follow-up data summarized in Table 1; their mean follow-up time was 18.9 months (range: 2.6–51 months).

## 3 Conclusion

We report here a rare case of LPL initially presenting with CPA compression. LPL rarely involves the CPA. It is difficult to diagnose pre-surgically and is usually misdiagnosed. With this article, we aimed to both present this rare entity and to emphasize the importance of keeping this differential diagnosis in mind when analyzing CPA lesions.
